# Thoracic horns on males of an urban dung beetle conform to a pattern of sigmoid allometry on the arithmetic scale

**DOI:** 10.1093/aesa/saaf040

**Published:** 2025-12-17

**Authors:** Gary C Packard

**Affiliations:** Department of Biology, Colorado State University, Fort Collins, CO, USA

A recent report in this journal described a pattern of biphasic, loglinear variation in length of the thoracic horn versus width of the prothorax (a proxy for body size) in males of the dung beetle *Onthophagus orpheus* (McCullough et al. 2025). The analytical protocol followed conventional practice for studies of bivariate allometry (Smith 1984): (1) transform the original measurements to logarithms, (2) plot the transformations on a bivariate graph, (3) fit some kind of linear model (ie, one with linear ordering of parameters) to the distribution, and (4) then interpret the outcome in the context of untransformed observations. In the present case, the fitted model was a 2-segment, piecewise linear regression, and the results were taken to mean that male beetles form 2 distinct subpopulations (morphs). One morph was said to be comprised of animals with relatively large bodies and horns (“Majors”), and the other was said to be comprised of beetles with relatively small bodies and horns (“Minors”). The results were then interpreted in the context of male–male combat and competition.

Despite the wide use of this protocol in contemporary research, the procedure actually does not follow good statistical practice, and investigations that adopt the protocol frequently yield results that are misleading or wrong (see Packard 2023 and references therein). Here, I present a new treatment of allometric variation in the size of horns on these dung beetles to explore implications of the prior analysis and to illustrate the power of a newer protocol that focuses on untransformed data.

## Implications of the Conventional Analysis

I downloaded measurements for the length of the horn and the width of the prothorax for 173 male beetles from the online supplement to the original investigation (McCullough et al. 2025). The measurements were transformed to logarithms (base 10) and plotted on a bivariate graph (not shown). Straight lines then were fitted to observations falling above and below the breakpoint of 0.545 in the piecewise regression fitted by McCullough et al. (2025). Values for slopes of the lines agreed with those reported in the original investigation.

**Fig. 1.**
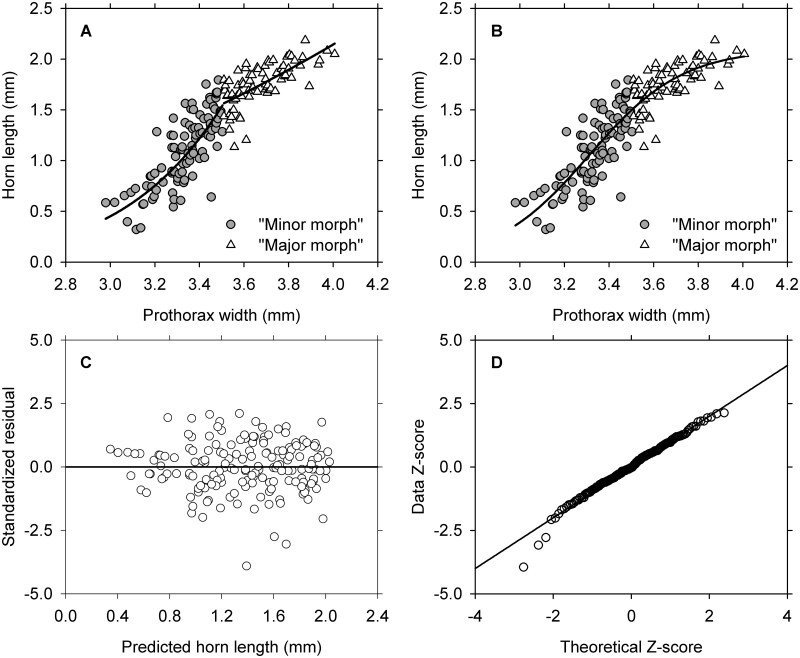
A) The straight lines fitted to transformations in the original investigation provide a way to estimate parameters in a pair of 2-parameter power functions describing the untransformed distribution. The equation describing putative “Minors” is *Y* = 0.0001 X^7.95^; that for “Majors” is *Y* = 0.08 X^2.41^. B) A 3-parameter sigmoid function with normal, heteroscedastic error provides a good visual description for the entire distribution. C) Standardized residuals for the sigmoid function were computed by dividing raw residuals by the root mean square for error. The residuals are randomly distributed with respect to the predicted values for the length of horns. D) Three odd values are apparent in the lower tail of the *Q*–*Q* plot, but the remaining 170 values correspond closely with expectations for a normal distribution.

The fitting of a straight line to logarithmic transformations is merely the first step in estimating parameters for a 2-parameter power function on the linear scale (Packard 2023). I accordingly back-transformed the equations and displayed the resulting functions on a bivariate graph with the untransformed observations (Fig. 1A). Each of the simple power equations provides a good visual description for the pattern over the limited range in body size to which the equation applies. However, 2 equations are needed to describe the pattern in what appears to be a single, curvilinear distribution.

## New Analysis on Untransformed Data

A shortcoming with the original analysis is that the data seem never to have been examined on the untransformed scale, despite the implied goal of characterizing and understanding the relationship between horn length and prothorax width in male beetles. My next step, consequently, was to use the Model Procedure in SAS 9.4 to fit several plausible models directly to untransformed observations by Full Information Maximum Likelihood (also see Endnote). Six models assumed lognormal, heteroscedastic error (like that which is obtained by back-transforming a line fitted to logarithms). Another 6 models assumed normal, homoscedastic error (ie, the error structure in standard regression protocols), and a final 6 assumed normal, heteroscedastic error (Packard 2023). Two models in each group of 6 were power functions (with and without an explicit, non-zero intercept), 2 were straight lines (again, with and without a non-zero intercept), and 2 were sigmoid functions (with and without a non-zero intercept). Thus, a pool of 18 statistical models was submitted to numerical analysis.

The assumption of constant variance (σ^2^) in each model was evaluated by performing a Breusch–Pagan test and by plotting standardized residuals against predicted values for the length of the horn. The assumption of normal distribution for residuals was evaluated by creating a *Q*–*Q* plot to compare raw residuals with a theoretical (normal) distribution. And finally, the models were evaluated with Akaike’s Information Criterion, or AIC (Burnham and Anderson 2002).

The numerical analysis summarized in Supplemental Table 1 revealed that the only acceptable models in the pool of candidate models were the sigmoid equations that assume normal, heteroscedastic error. Moreover, the 4-parameter sigmoid model is not substantially better than the 3-parameter model and consequently can be dismissed on grounds of overfitting (Burnham and Anderson 2002). Thus, the best model in the pool of candidate models is the 3-parameter sigmoid function with normal, heteroscedastic error.

Summary plots of the data are revealing. Figure 1B shows that the bivariate observations follow a curvilinear path that is well described by the 3-parameter sigmoid function. The untransformed data form a continuous distribution showing no indication of distinctive minor and major morphs. The plot of standardized residuals reveals 3 slightly unusual values (below −2.5), but the overall pattern of variation for the remaining 170 observations confirms that the variance assumption (constant σ^2^) was satisfied (Fig. 1C). Finally, the *Q*–*Q* plot for normal distribution for residuals is quite good, with most observations falling on or near the diagonal line characterizing a normal distribution (Fig. 1D).

To summarize, the bivariate plot of untransformed data reveals a distribution that is described quite well by a single statistical model, namely, a 3-parameter sigmoid function with normal, heteroscedastic error. The absence of a pattern in the distribution for residuals further supports the notion that the population of males is comprised of a single, but variable, morph.

## Discussion

The analytical protocol applied in the original investigation of horns on male *Onthophagus orpheus* was flawed by three mistakes that commonly occur in studies of allometry:

1. *The original, untransformed data were not examined graphically at the outset of the analysis.* Such a preliminary, graphical examination of the data is necessary to provide the investigator with first impressions concerning patterns in the bivariate distribution and thereby suggest, in turn, the kind of statistical model that will be needed to describe the distribution (Chatfield 1985).
2. *The data were transformed to logarithms when it was not necessary to do so.* Data transformation is meant to create a new mathematical distribution that comes closer than the original distribution to satisfying assumptions of the analytical protocol (Tukey 1957). However, all the assumptions that commonly are used to justify transformation in allometry actually can be satisfied in the arithmetic domain by changing the functional form for the descriptive equation and/or by changing the form for random error (Packard 2023). Logarithmic transformation also creates a new mathematical distribution that is difficult to evaluate correctly (Menge et al. 2018). Data are most likely to be interpreted correctly when they are analyzed on the original scale (Finney 1989).
3. *The equations fitted to logarithms in the original investigation were not validated on the scale of measurement*. No analysis is complete until the findings are validated graphically (Anscombe 1973), and this is best accomplished on the arithmetic scale (Finney 1989; Menge et al. 2018).

The current treatment of data for male *Onthophagus orpheus* avoids all three of these problems. So, how are the conclusions from the original study affected?

First and foremost, the pattern of allometric variation in length of the thoracic horn is continuous and sigmoidal, that is, not discontinuous or biphasic (Fig. 1B). This pattern of variation has been recorded for several other dung beetles (Emlen 1994; Moczek et al. 2004), so the observed pattern of allometric variation is not unique.

And second, the earlier characterization of the distribution as one of dimorphism was an overreach because the distribution is a continuous one with no break or interruption. There is little doubt that the relative size of the horn is greater in large beetles than in small ones, but continuity of intermediate morphs belies the notion of a dimorphism. The males are merely polymorphic with respect to relative size of the thoracic horn.

## Endnote

One referee expressed concern that the SAS routine fits a Model 1 regression instead of Model 2. Model 1 regression is based on the assumption that the response variable is measured with error while the predictor variable is measured without error. In contrast, Model 2 assumes that both the predictor and the response are measured with error. Relative merits of Model 1 versus Model 2 have been widely debated, largely (but not exclusively) in the context of straight lines fitted to logarithmic transformations of the original measurements (Kilmer and Rodríguez 2017). The question of Model 1 versus Model 2 is not a major concern in the current investigation, because the numerical analysis presented here serves only to confirm the pattern that is evident in a bivariate graph displaying untransformed measurements (Anscombe 1973).

## Supplementary Material

saaf040_Supplementary_Data
