# Supplementary material for: Thoracic horns on males of an urban dung beetle conform to a pattern of sigmoid allometry on the arithmetic scale
Source: Ann Entomol Soc Am. 2025 Dec 17;119(2):72–4. doi: 10.1093/aesa/saaf040 (PMC13017565; doi:10.1093/aesa/saaf040)
Supplement: saaf040_Supplementary_Data [file saaf040_supplementary_data.pdf]

Supplemental Table 1. Models fitted by linear and nonlinear regression to untransformed data for length of the thoracic horn vs width of the prothorax on male dung beetles

Predictive equation	Variance test (P)	Log-likelihood	Fitted parameters	AIC	Δ AIC
**Lognormal, heteroscedastic error**					
Straight line, zero intercept: $Y = 0.37\ X$	<0.001	−103.8	2	211.6	293.8
Straight line, non-zero intercept: $Y = -5.58 + 1.99\ X$	<0.001	−6.2	3	18.44	100.6
2-parameter power equation: $Y = 0.001\ X^{5.51}$	0.001	−17.7	3	41.4	123.6
3-parameter power equation: $Y = -5.04 + 1.65\ X^{1.09}$	<0.001	−6.2	4	20.4	102.6
3-parameter sigmoid equation: $Y = 1.29/(1 + \exp(-(X - 319.18)/ -0.83))$	<0.001	−127.1	4	262.2	344.4
4-parameter sigmoid equation:	<0.001	4.6	5	0.8	83.0

Y = 0.40 + 1.56/(1 + exp(−(X – 3.38)/0.14))					
**Normal, homoscedastic error**					
Straight line, zero intercept:<br>Y = 0.40 X	<0.001	−74.0	2	152.0	234.2
Straight line, non-zero intercept:<br>Y = −5.15 + 1.88 X	0.13	27.0	3	−48.0	34.2
2-parameter power equation:<br>$Y = 0.01\ X^{4.32}$	0.06	11.0	3	−16.0	66.2
3-parameter power equation:<br>Failed to converge on a solution	—	—	—	—	—
3-parameter sigmoid equation:<br>Y = 2.11/(1 + exp(−(X – 3.32)/0.21))	0.02	39.9	4	−71.8	10.4
4-parameter sigmoid equation:<br>Y = 0.30 + 1.73/(1 + exp(−(X – 3.36)/0.17))	0.03	40.6	5	−71.2	11.0
**Normal, heteroscedastic error**					

Straight line, zero intercept:<br><br>Y = 0.47 X	0.93	−52.6	3	111.2	193.4
Straight line, non-zero intercept:<br><br>Y = −4.76 + 1.77 X	0.99	28.9	4	−49.8	32.4
2-parameter power equation:<br><br>Biased parameter estimates	—	—	—	—	—
3-parameter power equation:<br><br>Failed to converge on a solution	—	—	—	—	—
3-parameter sigmoid equation:<br><br>Y = 2.10/(1 + exp(−(X – 3.31)/0.21))	0.94	46.1	5	−82.2	0
4-parameter sigmoid equation:<br><br>Y = 0.18 + 1.89/ (1 + exp(−(X – 3.35)/0.19))	0.94	46.3	6	−80.6	1.6

Variance was assessed with the Breusch–Pagan test. The count of fitted parameters includes variance. AIC is Akaike's Information Criterion.
